# Some Considerations About Cardiac Toxicity of Combination Therapy for Chronic Hepatitis C

**DOI:** 10.5812/hepatmon.8962

**Published:** 2013-05-14

**Authors:** Reza Karbasi-Afshar, Amin Saburi

**Affiliations:** 1 Cardiovascular Diseases Department, Baqiyatallah University of Medical Sciences, Tehran, IR Iran; 2 Chemical Injuries Research Center, Baqiyatallah University of Medical Sciences, Tehran, IR Iran; 3 Baqiyatallah Research Center of Gastroenterology and Liver Diseases (BRCGL), Baqiyatallah University of Medical Sciences, Tehran, IR Iran

**Keywords:** Hepatitis C, Interferon-Gamma, Ribavirin, Combined Modality Therapy

*Dear Editor,*

We read with great interest the recently published article by Almawardy et al entitled “Is Combination Therapy for Chronic Hepatitis C Toxic for Cardiac Function?” (1). They skillfully evaluated 120 cases with hepatitis C virus infections treated with a combination protocol consisting interferon gamma (INFg) a or b in addition to Ribavirin and their aimed to evaluate cardiac toxicity of this regiment during six months treatment. They finally concluded that “combination therapy does not cause a significant deterioration in cardiac function in patients with a chronic hepatitis C infection, and it may be used safely in patients without cardiac disease.” It seems that there are some remarks in this paper that it is better to consider.

1) There are various reports about cardiac disorders in patients with hepatitis C. It seems that direct cardiac toxicity of hepatitis C virus can confound the result of this study (2-4). The aim of this study was “to evaluate the effects of combination therapy pegylated interferon and ribavirin on cardiac function in patients with a chronic hepatitis C infection.” It was necessary to eliminate the pure effect of hepatitis C virus on cardiovascular system (such as with adjusting viral load or sustained virological response) to discuss more precisely about combination therapy impacts on heart (5). It also stated that “Adverse effects of Interferon include a cardiac toxicity were reported.” It is better to assessed cardiac toxicity of hepatitis C combination protocol in 3 groups including 1- INFg alone, 2-Ribavirin alone, 3- combination of them. It is possible that there are some synergistic or additive effects between INFg and Ribavirin (6).

2) They assessed these patients’ total cardiovascular health using “detailed medical history, thorough clinical examination, 12 lead electrocardiogram (ECG), and echocardiography” but none of them are sufficient for assessment of coronary artery diseases which was frequently reported in these patients (7). Moreover, cardiac biomarkers such as CRP, troponin, serum N-terminal pro-B-type natriuretic peptide are more valid markers for predicting cardiovascular abnormalities than ECG and echocardiography show established heart disorders (8, 9). Also, is all echocardiographic studies were performed by one cardiologist or not? The results of echocardiography depend on operators. Anti-HCV therapy can induce reversible autonomic dysfunction may be caused by the immunomodulatory actions of interferon alfa-2 (10). Autonomic dysfunction can affect cardiovascular health was not considered in this report.

3) One of most important cardiovascular complications of viral hepatitis therapy is ventricular dysfunction and so cardiac exercise test or myocardial perfusion scintigraphy is more effective test than ECG or even echocardiography at early stage? (2, 3). Moreover, how the authors could rule out the Cardiomyopathy while it’s a complication of pegylated interferon and ribavirin? (11).

4) The adverse effects of treatment can be present after 6 months follow up, therefore six months follow up may be not sufficient (10).

5) About statistical analysis; 1- for comparison of quantitative values between before and after treatment, the “Wilcoxon test” was used although McNemmar test is more suitable. 2- It is better to perform regression model for variables with 0.05 < P < 0.2 such as all items in table-2. 3- Power of study was not estimated for insignificant items to rule out the insufficient sample size. Regarding to the “Tabulation Table”, it seems that Pvalue can be significant if the sample size was raised. 

6) Is there any significant difference in serum levels of lipid profiles as cardiovascular disorders risk factors during the study? Moreover, the previous reports stated that acute hepatitis C infection lowers serum lipid levels (12). Also, the mean amount of fasting blood sugar was as equal as 109.1 ± 34.58 mg/dl which indicate that some patients were diabetic or at least IFG (Impaired fasting glucose). Diabetic status can predispose patients for cardiovascular attacks and this can effect on the results. On the other hand, Anti Hepatitis C virus treatment can affect insulin resistance which is important in diabetic patients with viral hepatitis (13). Are these patients controlled for physical activity, diet and other affecting factors? (14).

7) These medications can effect on other organs and tissue such as thyroid gland, blood pressure and etc which can influence cardiovascular health (13, 15). Therefore, we cannot carefully conclude about the safety of these drugs on heart while we did not consider their effects on other organs.

8) Viral load and other properties of disease can affect cardiovascular system (16). On the other hand, sustained virological response may associate with these properties. Therefore, it seems that for more accurate conclusion we should assess a probable correlation between these viral properties of hepatitis C infection during the treatment and cardiovascular health indexes.
